# Relative Number and Distribution of Murine Hypothalamic Proopiomelanocortin Neurons Innervating Distinct Target Sites

**DOI:** 10.1371/journal.pone.0025864

**Published:** 2011-10-04

**Authors:** Connie M. King, Shane T. Hentges

**Affiliations:** Department of Biomedical Sciences, Colorado State University, Fort Collins, Colorado, United States of America; The Research Center of Neurobiology-Neurophysiology of Marseille, France

## Abstract

Proopiomelanocortin (POMC) neurons send projections widely throughout the brain consistent with their role in regulating numerous homeostatic processes and mediating analgesia and reward. Recent data suggest that POMC neurons located in the rostral and caudal extents of the arcuate nucleus of the hypothalamus may mediate selective actions, however it is not clear if POMC neurons in these regions of the arcuate nucleus innervate specific target sites. In the present study, fluorescent microspheres and cholera toxin B were used to retrogradely label POMC neurons in POMC-DsRed transgenic mice. The number and location of POMC cells projecting to the supraoptic nucleus, periaqueductal gray, ventral tegmental area, paraventricular nucleus, lateral hypothalamic nucleus, amygdala and the dosal vagal complex was determined. Tracer injected unilaterally labeled POMC neurons in both sides of the arcuate nucleus. While the total number of retrogradely labeled cells in the arcuate nucleus varied by injection site, less than 10% of POMC neurons were labeled with tracer injected into any target area. Limited target sites appear to be preferentially innervated by POMC neurons that reside in the rostral or caudal extremes of the arcuate nucleus, whereas the majority of target sites are innervated by diffusely distributed POMC neurons. The modest number of cells projecting to each target site indicates that relatively few POMC neurons may mediate potent and specific physiologic responses and therefore disturbed signaling in a very few POMC neurons may have significant consequences.

## Introduction

Hypothalamic POMC neurons have been the focus of much investigation recently due to their ability to inhibit food intake and reduce body weight [1]. POMC neurons primarily affect energy balance through the release of the peptide alpha-melanocyte stimulating hormone (α-MSH) although these neurons also release other melanocortins and the opioid beta-endorphin (β-end) which can also affect energy balance [1], reward and analgesia [2], [3]. In addition to the peptides cleaved from the POMC prohormone, POMC neurons can also express several other peptide and non-peptide transmitters including nociceptin [4] cocaine-amphetamine-regulated transcript [5], acetylcholine [6], GABA [7], [8] and glutamate [8], [9] adding to the ability of these neurons to regulate a wide range of physiologic processes.

Fibers and axon terminals immunoreactive for POMC peptides have been detected throughout the brain and are particularly dense in areas involved in general homeostasis such as hypothalamic and brainstem nuclei, as well as areas that mediate reward (e.g. ventral tegmental area, stria terminalis, nucleus accumbens and lateral hypothalamus) [10], [11], [12], [13]. POMC-containing fibers are also present in the amygdala, periaqueductal grey and various other structures [10], [11], [12], [13]. Although there is a small group of POMC neurons in the nucleus of the solitary tract (NTS) [14], [15] lesion and tract tracing studies indicate that hypothalamic POMC neurons are a primary source of POMC-peptide immunoreactive fibers [15], [16], [17]. The physiologic actions of NTS-derived POMC peptides are not are not well defined primarily for technical reasons, including a low number of POMC neurons in the region (∼200 POMC in the mouse NTS compared to >3,000 in the arcuate nucleus) [18] and that POMC peptides are difficult to detect in neuronal cell bodies in NTS using immunohistochemical methods [18], [19]. Although there are local brainstem projections from POMC somas in the NTS [20], the majority of POMC peptide immunoreativity in the hypothalamus, forebrain and even the NTS arises from hypothalamic POMC neurons [15], [20], [21].

The actions of POMC peptides are dependent on the array of receptors expressed at specific sites that the neurons innervate as well as the connectivity and output of downstream target sites. Therefore, blocking the actions of POMC-derived peptides at very specific target sites can have distinct physiologic consequences. For example, recent work demonstrates that α-MSH acts on its receptors in the paraventricular nucleus (PVN) to inhibit food intake [22], but acts on neurons outside of the PVN to affect metabolism [23].

In addition to a single POMC peptide having diverse actions at specific target sites, individual POMC neurons in the hypothalamus can vary in the compliment of transmitters that they express [8], [24] and subpopulations of POMC neurons respond differently to regulatory factors such as steroid hormones and metabolic signals including leptin and insulin [25], [26]. Selective regulation and heterogeneity in transmitter phenotype often correlate well with the cell's location within the rostral-caudal and/or medial-lateral extent of the arcuate nucleus of the hypothalamus [8], [27], [28]. Thus, the present experiments used retrograde tract tracing to address the hypothesis that POMC neurons residing in different areas of the arcuate nucleus innervate discrete target sites. The results indicate that retrograde tracer injected into any of the 8 target sites tested labeled ∼10% or less of POMC neurons. Considerable variability in the rostral/caudal distribution of POMC cells innervating select target sites was noted and the dorsal vagal complex appears to receive projections from POMC neurons in the rostral portion of the arcuate nucleus selectively. Altogether the data provide a comprehensive analysis of hypothalamic POMC neuron projections to a wide range of target sites.

## Methods

### Animals

Male mice expressing the fluorescent protein discoma red (DsRed) under the control of the *Pomc* promoter (POMC-DsRed) were used for all experiments. All mice were 8 weeks old at the time of tracer injection. The mice were produced by standard techniques as described previously [8] and backcrossed onto the C57BL/6J genetic background. The reliable expression of the DsRed transgene in authentic POMC neurons was previously demonstrated [8] and the transgene does not appear to affect the health or basal electrical properties of POMC neurons according to earlier studies [8], [29]. Mice were housed at controlled temperature (22–24°C) with a 12 h light/dark cycle and were given standard rodent chow and tap water *ad libitum*. All animal procedures met United States Public Health Service guidelines as outlined in the Guide for the Care and Use of Laboratory Animals of the National Institutes of Health and were approved by the Institutional Animal Care and Use Committee at Colorado State University (approval number 10-1915).

### Stereotaxic microinjection

Fluorescent microspheres (0.04 µm, Invitrogen) or AlexaFluor-647 conjugated-cholera toxin subunit B (CTB, Invitrogen) was microinjected unilaterally (unless otherwise indicated) into target sites of anesthetized (2–5% isofluorane) mice under aseptic conditions using a small animal stereotax (Kopf Instruments). Animal heath was monitored throughout all procedures and all efforts were made to minimize distress. All injections were 200 nl (except where noted) and were delivered via a 25 gauge (internal diameter, 0.13 mm; outer diameter, 0.483 mm) Hamilton syringe over 2 min. The needle was left in place for >3 min before being withdrawn slowly over 2 min. The coordinates for injections were calculated using *The Mouse Brain in Sterotaxic Coordinates*, second edition by Paxinos and Franklin and adjusted as needed to the final coordinates as follows (mm from bregma, lateral, dorsal/ventral, respectively): supraoptic nucleus (−0.42, −1.5, 5.4); paraventricular nucleus (−0.94, −0.5, 5.5); lateral hypothalamus (−0.42, −1.3, 5); ventral tegmental area (−2.8, −0.24, 5.3); periaqueductal grey (−3.25, −0.04, 3.9); bed nucleus of the stria terminals (1.25, −0.98, 4.1); amygdala (0, −2.75, 5); and dorsal vagal complex (−3, −0.4, 4.15). The success rate for hitting the target site varied from ∼43% (dorsal vagal complex) to >70% (supraoptic nucleus and periaquedutal grey) depending on the size of the target and proximity to a ventricle.

### Tissue processing and imaging

5 d (unless otherwise noted) after injection, tissue was processed for imaging using standard techniques. Briefly, Mice were deeply anesthetized with sodium pentobarbital and perfused transcardially first with a 10% sucrose solution, then with 4% paraformaldehyde in 0.1 M potassium phosphate buffer. Brains were post-fixed 18 h at 4°C in 4% paraformaldehyde and sectioned at 50 µm on a vibratome. Sections were mounted in the order sliced onto poly-lysine-coated slides and cover-slipped with aqueous mounting media (Polysciences, Inc, Aqua/Poly Mount). Imaging was performed on a Zeiss 510 Meta confocal microscope. Green fluorescent microspheres were imaged using a 488/543 nm bandpass filter and emission was detected using a 505/530 nm bandpass filter, DsRed was imaged using a 488/543 nm bandpass excitation filter and a 585/615 nm bandpass emission filter and Alexa Fluor 647-conjugated CTB was detected using a 514/633 nm bandpass excitation filter and a 650 nm longpass emission filter. Images at each wavelength were collected sequentially to limit the possibility of crossover between channels. Three thin-plane images each 3 µm apart were merged into one stacked image to be used for cell counts. Approximately one-tenth of the total volume of the arcuate is included in the counting since a ∼10 µm stacked confocal image was produced for every-other 50 µm-thick section for counting. The exact z-position of the images collected varied based on the location of cells in the slice, but the most superficial ∼10 µm from either surface was not imaged to ensure collecting complete images and to avoid imaging where there may be surface damage from cutting and handling of the tissue. The location of each injection was verified in the fixed tissue by visualizing the dye at the injection site.

### Cell counting

For each brain sectioned, the hypothalamus was imaged such that images were collected every 50–100 µm from the retrochiasmatic area (rca) through the caudal extent of the arcuate (arc) nucleus. For data presentation and analysis, the area containing POMC-DsRed neurons was divided into 3 regions identified as rostral, mid and caudal arcuate nucleus with each division representing ∼450 µm of rostral/caudal length beginning at the most rostral POMC-DsRed neuron observed. Three or 4 images were collected through the rostral/caudal extent of each region. POMC-DsRed and tracer-containing cells were counted from the images displayed in Photoshop. Cells were marked during counting to avoid duplicate counts and to allow switching between channels to verify the presence or absence of co-labeling.

### Statistics

Data are presented as mean ± SEM. Statistical differences between 2 groups were analyzed using two-sided unpaired t-tests. Comparisons across different rostral-to-caudal divisions or among target sites were made using one-way ANOVA with Bonferonni post-hoc analyses. Data comparing tracer transport times were analyzed using a Pearson test for correlation. Groups were considered different if the p value was less than 0.05 with a 95% confidence interval.

## Results

### Effects of injection volume, transport time, tracer and accurate targeting on labeling

**Figure 1:**
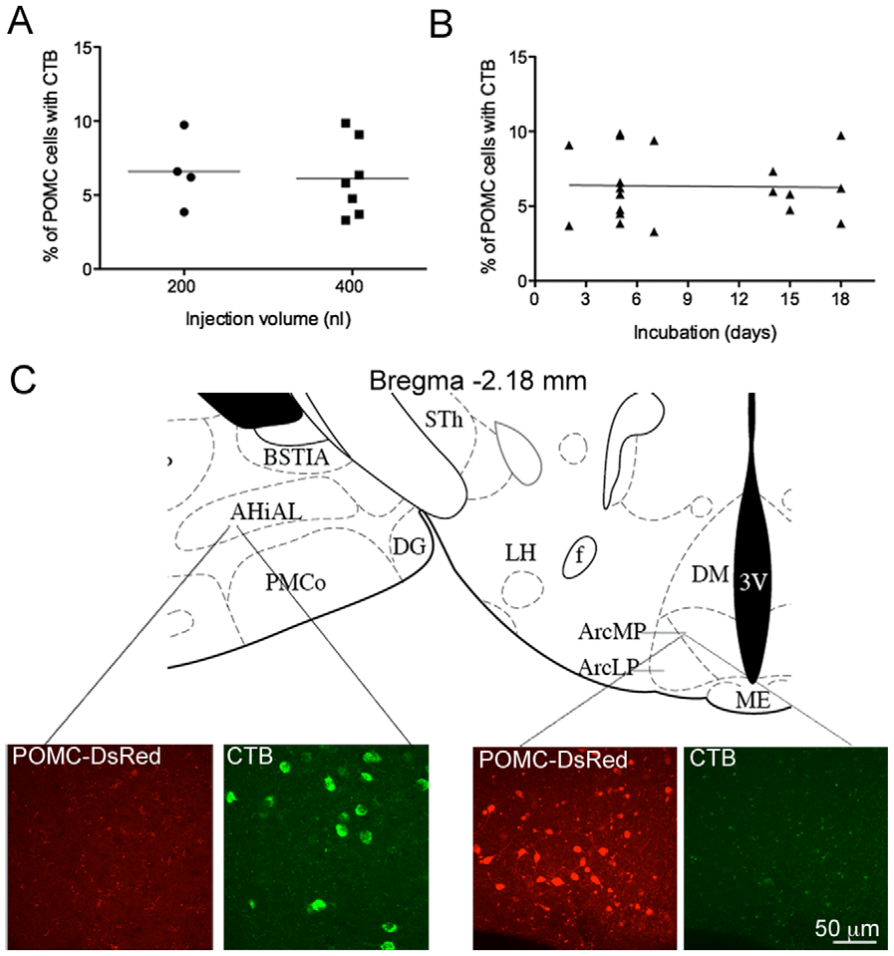
Effects of injection volume, transport time, and placement of injection. (A) Scatter plot indicating the percent of POMC-DsRed neurons containing CTB after 200 nl or 400 nl injections of CTB into the VTA. Each point represents the counts from an individual animal. (B) Plot indicating the percent of POMC-DsRed cells that contain CTB at various time-points after CTB was injected into the VTA. Each point represents the counts from an individual animal. (C) Images show POMC-DsRed cells (red) and CTB-positive cells (green) in the AHiAL (left) and the arcuate nucleus (right) after CTB was injected into the ventral striatum. There are no POMC-DsRed cells in the AHiAL and no CTB-positive cells in the arcuate nucleus. The diagram shown for reference was adapted from Paxinos and Franklin (2nd edition, © 2001). Abbreviations: BSTIA, bed nucleus of the stria terminalis- intraamygdaloid subdivision; AHiAL, amygdalohippocampal area- anterolateral; PMco, posteromedial cortical amygdaloid nucleus; DG, dentate gyrus; STh, subthalamic nucleus; LH, lateral hypothalamus; f, fornix; Arc, arcuate nucleus (MP, medial posterior; LP, lateroposterior); DM, dorsomedial hypothalamic nucleus; 3V, third ventricle; ME, median eminence.

Experiments were performed to determine the appropriate injection volume and transport time using injections into the VTA. Increasing the injection volume into the VTA from 200 to 400 nl did not affect the percent of POMC cells labeled (Figure 1A; difference of means 0.47%, 95% confidence interval −3.1 to 4.0, p = 0.76) nor the total number of labeled cells in the arcuate nucleus (difference of means 206, 95% confidence interval −51 to 463, p = 0.1 by unpaired t-test). There was also no difference in the percent of POMC neurons labeled with CTB when tissue was processed 2, 5, 7, 14 or 18 days after the injection (Figure 1B; p = 0.9 by Pearson correlation, r = −0.02, 95% confidence interval −0.47 to 0.43) suggesting that maximal labeling was achieved within 2 days.

Since different tracers may be taken into axon terminals in distinct ways with varying efficiency, an alternative retrograde tracer was also tested for its ability to retrogradely label POMC neurons. Fluorescent microspheres were injected into the VTA (200 nl) 5 d prior to tissue processing. The percent of POMC cells with fluorescent microspheres was not significantly different from the percent labeled using CTB (6.2±0.82% versus 5.2±1.2% for CTB and microspheres, respectively; p = 0.5, N = 5–6 by unpaired t-test). CTB was used for the majority of studies as it more completely labeled entire cells compared to the microspheres which resulted in diffuse punctate labeling (not shown) and thus, CTB-positive cells were somewhat easier to define.

CTB uptake into POMC neurons appears to be specific and dependent on axon terminals. When CTB was injected into the 3rd ventrical, the cortex, various parts of the striatum including nucleus accumbens, and very medial PVN, there were no POMC cells labeled with tracer in the arcuate nucleus. Figure 1C shows an example where the target was the rostral BST but the injection was slightly rostral in the nucleus accumbens/caudate/putamen. Although no cells in the arcuate nucleus were labeled with CTB, there was labeling in the amygdalohippocampal area (AHiAL). Often injections missing the target site were in areas with dense POMC-DsRed-positive fibers and yet POMC cells in the arcuate nucleus were not labeled with CTB. Therefore it seems unlikely that fibers of passage are a significant source of labeling in the arcuate nucleus.

Thus, based on the control experiments, all of the following experiments were performed using 200 nl CTB tracer with 5 d transport time with confidence that labeling was due to intact projection fibers.

### Projections from arcuate nucleus to various target sites

**Figure 2:**
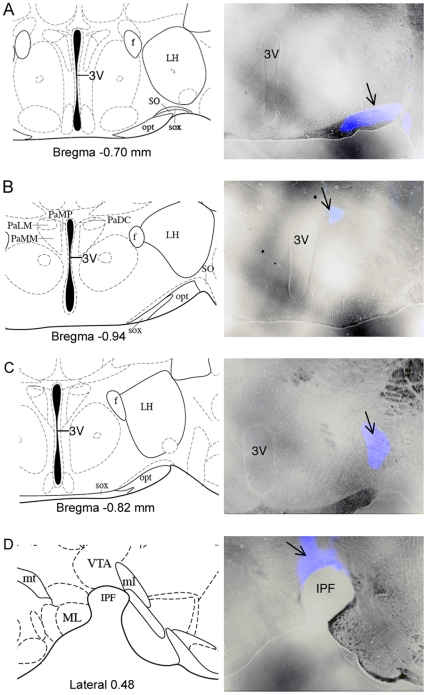
Placement of CTB injections. Bright-field and epifluorescent images were taken in the identical field and overlayed to produce the representative images in A–D (right side). The reference diagrams on the left side were adapted from the Mouse Brain Atlas of Paxinos and Franklin (2nd edition, © 2001). Coronal sections are shown for injections into (A) the supraoptic nucleus (SON), (B) the paraventricular nucleus (PVN) and (C) the lateral hypothalamic area (LH). An injection into the ventral tegmental area (VTA) is shown in a slice prepared in the sagittal plane (D). Abbreviations: f, fornix; 3V, third ventricle; LH, lateral hypothalamus; SO, supraoptic nucleus; opt, optic tract; sox, supraoptic decussation, PaLM, paraventricular hypothalamic nucleus-lateral magnocellular; PaMM, paraventricular hypothalamic nucleus-medial magnocellular; PaDC, paraventricular hypothalamic nucleus-dorsal cap; PaMP, paraventricular hypothalamic nucleus-medial parvicellular part; mt, mammillothalamic tract; ml, medial lemniscus; VTA, ventral tegmental area; IPF, interpeduncular fossa.

**Figure 3:**
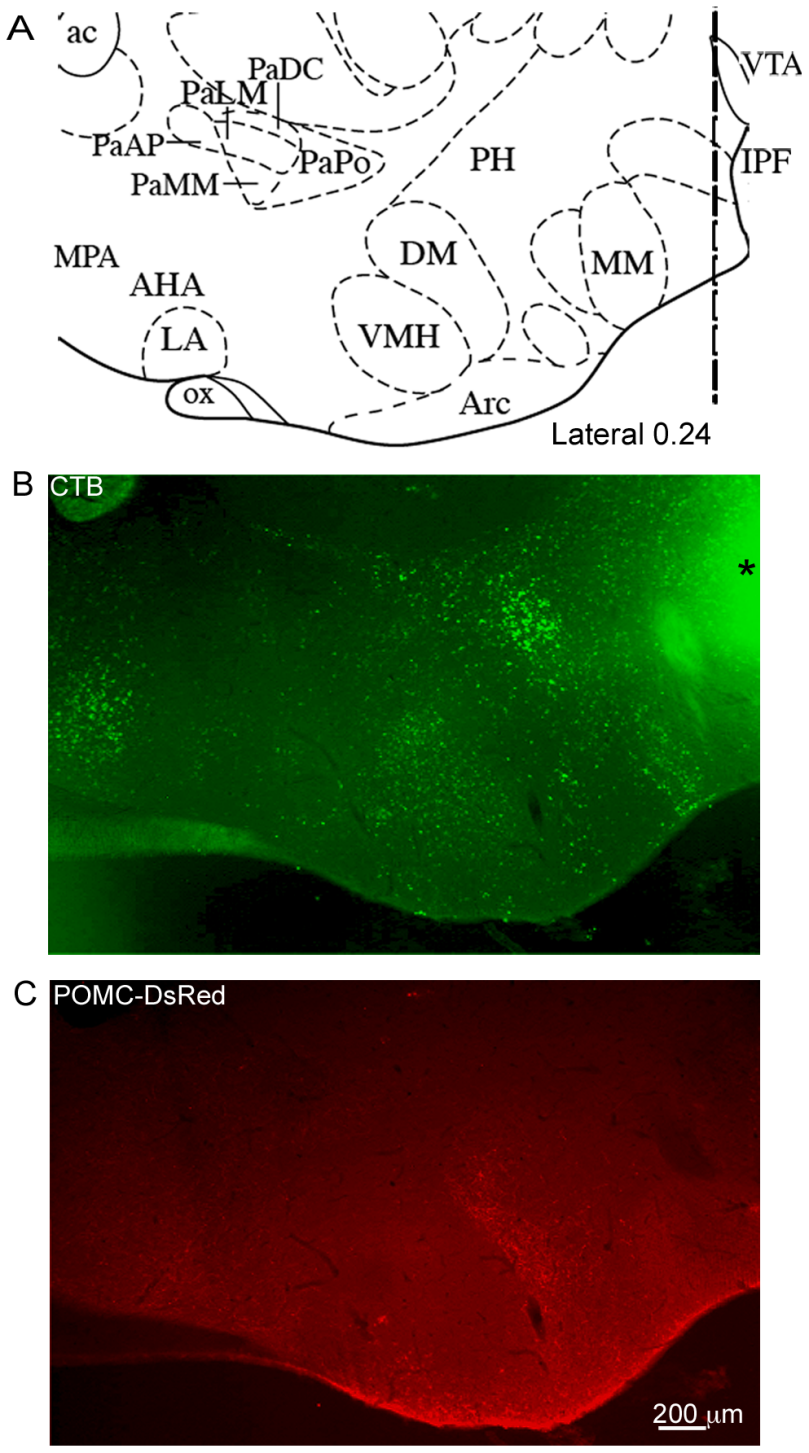
CTB selectively labels cell in distinct brain regions. (A) A portion of the atlas page (Lateral 0.24) from Paxinos and Franklin (2nd edition, © 2001) corresponding to the sagittal section shown in (B) and (C). The dotted line in (A) depicts where the images in (B) and (C) are cropped. CTB was injected into the VTA and a low-power image containing the arcuate nucleus was taken to reveal the distribution of CTB (B). Fluorescence from the injection site can be seen at the upper right edge of the photo in (B, marked with asterisk). (C) The same field of view as in (B) imaged to reveal the POMC-Ds-Red labeled cells in the arcuate and some of their prominent fibers rostral and caudal to the arcuate nucleus. Abbreviations: ac, anterior commissure; PaAP, paraventricular hypothalamic nucleus-anterior parvocellular; PaLM, paraventricular hypothalamic nucleus-lateral magnocellular; PaMM, paraventricular hypothalamic nucleus-medial magnocellular; PaDC, paraventricular hypothalamic nucleus-dorsal cap; PaPo, paraventricular hypothalamic nucleus-posterior; PH, posterior hypothalamic area; MPA, medial preoptic area; AHA, anterior hypothalamic area; LA, lateroanterior hypothalamic nucleus; VMH, ventromedial hypothalamic nucleus; DM, dorsomedial hypothalamic nucleus; Arc, arcuate hypothalamic nucleus, MM, medial mammilary nucleus; IPF, interpeduncular fossa; OX, optic chiasm; VTA, ventral tegmental area.

Retrograde tracer was injected into distinct brain regions innervated by hypothalamic neurons. The location of each injection was verified in fixed sections using low-power bright-field microscopy to visualize the tissue combined with epifluorescence to detect the injection site (some example images are shown in Figure 2). Any animals where the injection site could not be positively confirmed were omitted from further analysis unless used as a control for off-target injections. CTB traveled from the injection site to cell bodies in distinct regions throughout the brain in a manner that varied by the site injected. For example, CTB injected into the ventral tegmental area (VTA) labeled cells in the arcuate nucleus and also heavily labeled cells in the posterior hypothalamus, ventral medial nucleus of the hypothalamus and the preoptic area (Figure 3).

**Figure 4:**
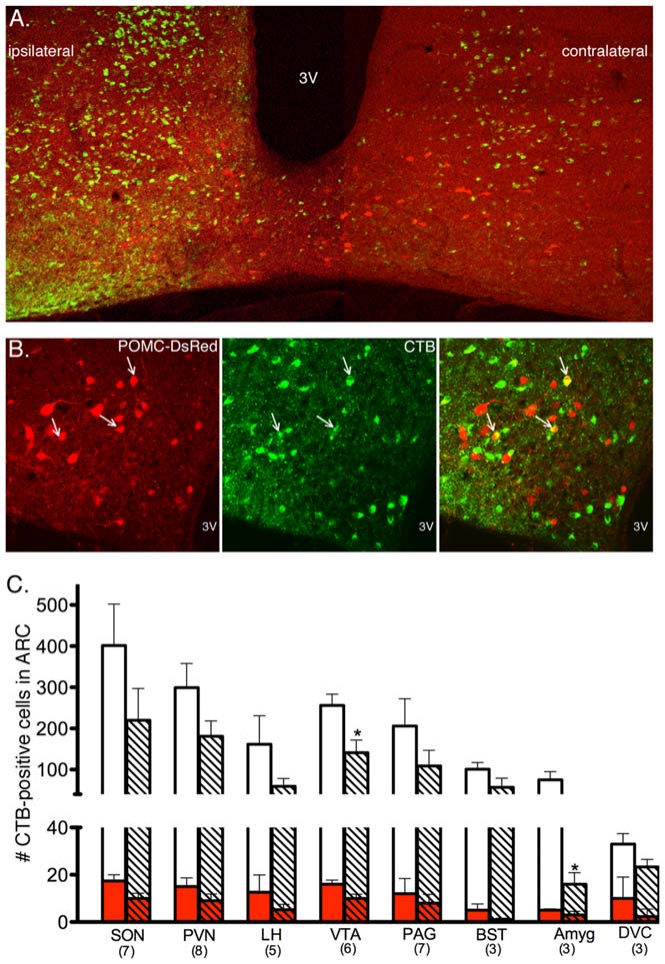
CTB labels many non-POMC cells and few POMC cells. (A & B) Example images after CTB injection into the SON. (A) Low-power image in the arcuate nucleus shows POMC neurons (red) and CTB-containing neurons (green). The side of the arcuate corresponding to the side of injection (ipsilateral) has more intense CTB labeling compared to the contralateral side. (B) Higher magnification image taken in the ipsilateral side shows POMC neurons with (white arrows) and without CTB label. Many neurons contain CTB, but not POMC-DsRed. 3V = 3rd ventricle. (C) Graph showing the number of cells imaged that have CTB after injections into the various target sites (white bars). The number of CTB-positive cells that also express POMC-DsRed are shown in the red portion of the bars. Solid bars correspond to counts from the side of the arcuate nucleus ipsilateral to the injection; striped bars correspond to the contralateral side. Data are presented as mean ± SEM. The numbers in parentheses indicate the number of animals in each group. The asterisks denote p<0.05 compared to ipsilateral side for same injection target. Abbreviations: SON, supraoptic nucleus; PVN, paraventricular nucleus; LH, lateral hypothalamic area; VTA, ventral tegmental area; PAG, periaqueductal gray; BST, bed nucleus of the stria terminalis; Amyg, amygdala; DVC, dorsal vagal complex.

The number of labeled cells in the arcuate nucleus was determined for each injection site. Injections into the supraoptic nucleus (SON) resulted in the greatest total labeling in the arcuate nucleus (ARC). Injections into the paraventricular nucleus (PVN), lateral hypothalamus (LH), VTA, and periaqueductal grey (PAG) also labeled many cells (Figure 4C). A modest number of cells were labeled from the bed nucleus of the stria terminalis (BST) and amygdala. Injections of tracer into the dorsal motor nucleus of the vagus (10N) or more broadly- the dorsal vagal complex (DVC), labeled very few neurons in the arcuate nucleus (Figure 4). Overall, there was significant variability in the number of cells labeled when CTB was injected into the 8 different sites (p = 0.02 by 1-way ANOVA). Labeling was evident both ipsilateral and contralateral to the injection (Figure 4A & C). Although fewer cells were labled contralateral to the injection, only the VTA and amygdala had significantly less labeling on the contralateral side (p = 0.02 and p = 0.05 for VTA and amygdala, respectively using unpaired t-tests).

### Number of POMC neurons with retrograde label

**Figure 5:**
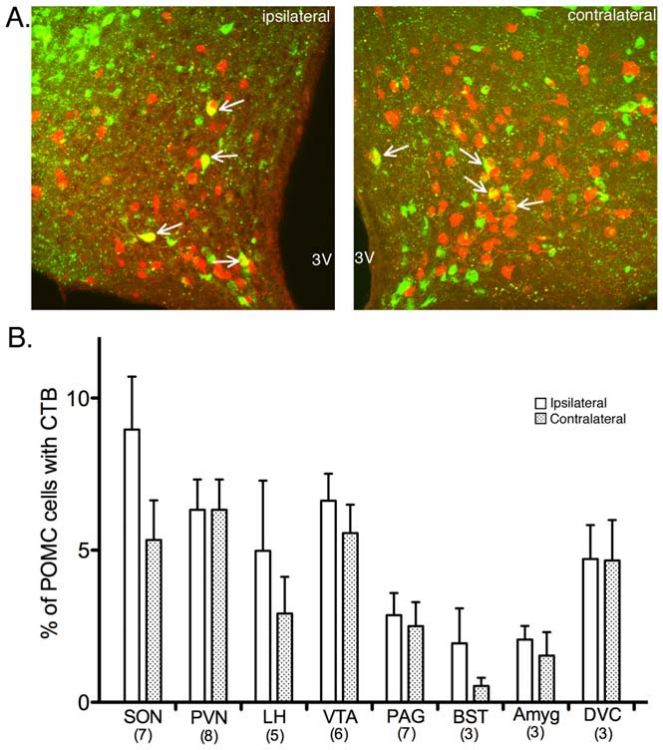
The percent of POMC neurons retrogradely-labeled with CTB varies by injection site. (A) Example images showing POMC-DsRed cells (red) and CTB-containing cells (green) in the arcuate nucleus after CTB was injected into the PVN. The percent of POMC-DsRed cells that contain CTB is similar on the side of the arcuate nucleus corresponding to the side receiving the injection (left picture) and the side opposite the injection (right picture). Arrows indicate POMC-DsRed cells that contain CTB. (B) Bar graph representing the percent of POMC-DsRed cells labeled with CTB in the arcuate nucleus after CTB was injected into the indicated target sites. There was a significant difference in labeling across target sites (p<0.001); no statistical differences were detected between the ipsilateral and contralateral sides for any injection site. Numbers in parentheses indicate the number of animals in each group. All data are mean ± SEM.

POMC neurons represent only a small subset of neurons in the arcuate nucleus. Therefore, although a significant number of cells in the arcuate nucleus were labeled with CTB, only a small number of those were POMC neurons (Figure 4, inset red bars). Between 1.7 and 7.9% of CTB-positive cells expressed POMC-DsRed for all injection sites except DVC where 46% of the CTB-containing cells were POMC neurons. The percentage of POMC cells containing CTB varied significantly by injection site (p<0.01 by one-way ANOVA) in both the ipsilateral and contralateral sides of the arcuate nucleus (Figure 5). There was not a statistically significant difference in the percent of POMC cells labeled on the ipsilateral or contralateral side of the arcuate nucleus for any injection site (SON, difference of means 3.6±2.2% (95% confidence interval −1.1 to 8.3), p = 0.12; PVN, difference of means 0±1.4% (95% confidence interval −3.0 to 3.0), p = 1; LH, difference of means 2.1±2.6% (95% confidence interval −3.4 to 8.1), p- 0.45; VTA, difference of means 1.1±1.3% (95% confidence interval −1.8 to 3.9), p = 0.43; PAG, difference of means 0.36±1.1% (95% confidence interval −2.0 to 2.7), p = 0.74; BST, difference of means 1.4±1.2% (95% confidence interval −1.9 to 4.7), p = 0.3; amygdala, difference of means 0.53±0.44% (95% confidence interval −1.9 to 3.0), p = 0.58; and DVC, difference of means 0.05±1.7% (95% confidence interval −4.8 to 4.9), p = 0.98).

### Location of POMC neurons labeled with CTB

**Figure 6:**
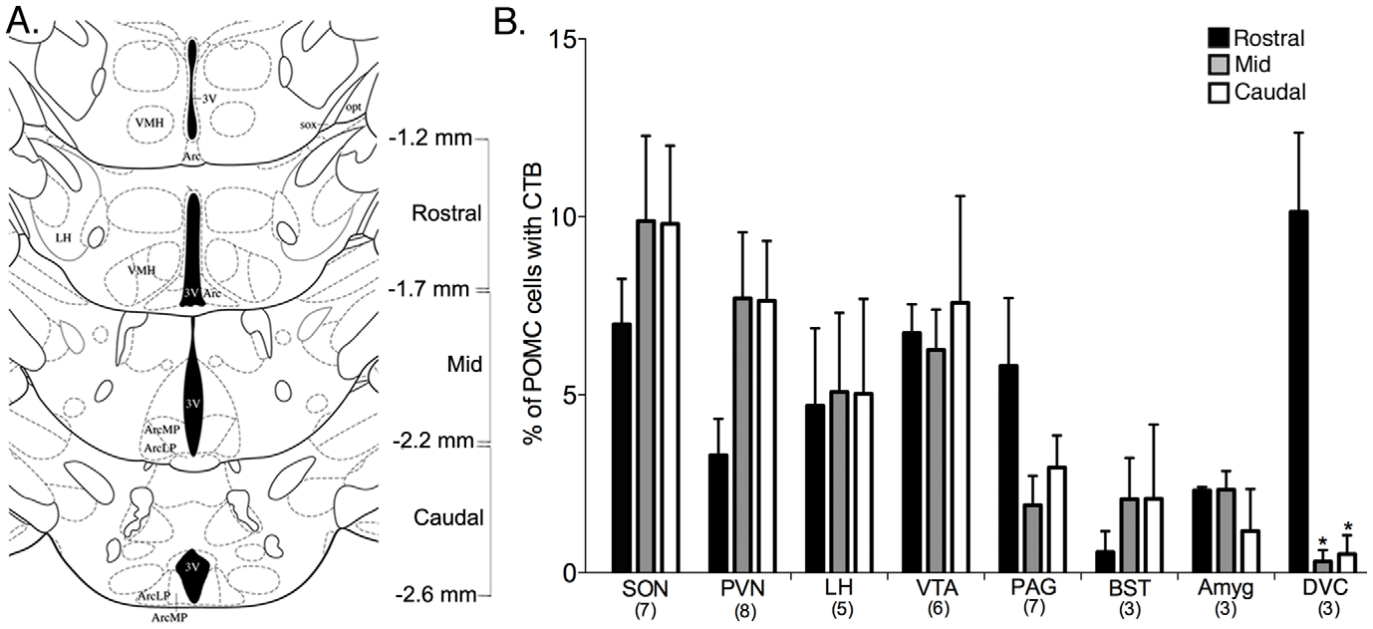
Rostral-to-caudal distribution of POMC neurons that project to distinct target sites. (A) Atlas diagrams modified from Paxinos and Franklin (2nd edition, © 2001) depicting the cut-offs used to define rostral, mid and caudal regions of the arcuate nucleus. Values are distance from Bregma. (B) Bar graph showing the percent of POMC-DsRed cells that contained CTB in the rostral, mid and caudal portions of the arcuate nucleus after CTB was injected into various target sites. Numbers in parentheses indicate the number of animals in each group. Asterisks indicate p<0.05 compared to rostral sections for that injection site. All data are mean ± SEM. Abbreviations: VMH, ventromedial hypothalamic nucleus; 3V, third ventricle; Arc, arcuate nucleus (MP, medial posterior; LP, lateroposterior); sox, supraoptic decussation; opt, optic tract; LH, lateral hypothalamus.

To determine if POMC cells located along the rostral-to-caudal extent of the arcuate nucleus may differentially project to certain target sites, brain sections from rostral, mid and caudal regions of the arcuate nucleus (as depicted in Figure 6A) were analyzed separately. CTB injected into the PAG and DVC preferentially labeled a higher portion of POMC neurons in the rostral-most region of the arcuate nucleus (statistically significant only for the DVC, p = 0.003 by one-way ANOVA). Tracer injected into the LH, VTA or amygdala labeled cells evenly throughout the arcuate nucleus. When CTB was injected into the SON, PVN and BST, fewer POMC cells labeled with CTB in the rostral portion of the arcuate nucleus as compared to the mid and caudal regions (Figure 6B) although there were no statistically significant differences in CTB labeling alone throughout the arcuate nucleus for any injection site except for the DVC (p = 0.009 by one-way ANOVA and see table 1) where there is more CTB labeling overall in the rostral portion of the arcuate nucleus (46% of which is in POMC neurons). Differences between medial and lateral portions of the arcuate nucleus were not apparent.

**Table 1:** Percent of POMC cells with CTB compared across rostral to caudal regions.

Areas Compared	SON	PVN	LH	VTA	PAG	BST	Amyg	DVC
Rost v. Mid	−2.9 (−10.4 to 4.6)	−4.4 (−10.0 to 1.2)	−0.4 (−9.7 to 8.9)	0.5 (−6.8 to 7.7)	3.9 (−1.0 to 8.8)	−1.5 (−8.1 to 5.1)	−0.02 (−2.7 to 2.7)	9.8 * (3.6 to 16)
Rost v. Caud	−2.8 (−10.4 to 4.7)	−4.3 (−10.1 to 1.5)	−0.4 (−9.6 to 8.9)	−0.9 (−8.1 to 6.4)	2.9 (−2.3 to 8.0)	−1.5 (−8.1 to 5.1)	1.1 (−1.9 to 4.1)	9.6 * (3.4 to 15.8)
Mid v. Caud	0.9 (−7.4 to 7.6)	0.07 (−5.7 to 5.9)	0.05 (−9.2 to 9.3)	−1.3 (−8.6 to 6.0)	−1.1 (−6.2 to 4.0)	−0.2 (−6.6 to 6.6)	1.2 (−1.9 to 4.2)	−0.2 (−6.4 to 6.0)

Mean difference for the percent of POMC cells that contain the retrograde tracer (CTB) between rostral (Rost), middle (Mid) and caudal (Caud) regions of the arcuate nucleus with 95% confidence intervals in parentheses for tracer injections into each of the indicated target sites. Asterisks denote P<0.01.

## Discussion

The number and range of functions attributed to POMC neurons and their transmitters underscores the importance of better understanding the circuitry associated with these neurons. Although many studies have examined output from the arcuate nucleus [10], [30], few have described outputs specifically from POMC neurons [31]. Additionally, it is difficult to relate information between different tracing studies due to differing methods, tracers, and ways of reporting the results. By using consistent methods and analyses for several target areas in the same study, the present results represent a comprehensive analysis of relative innervation to differing target sites and the location of POMC cells that project to specific target sites. The results indicate that only a small fraction (∼10% or less) of POMC neurons projects to any one target area although there are some distinctions in whether POMC neurons in the rostral or caudal portions of the arcuate nucleus are more likely to project to specific target areas.

### Hypothalamic targets of POMC neurons

POMC neurons innervate many regions of the hypothalamus including the PVN [32], LH [33] and SON [34]. The results here provide additional support for POMC terminals in these areas, however they also indicate that only a modest percent of POMC neurons project to each of these hypothalamic areas (Figure 5). Functional studies provide strong evidence that POMC peptides, particularly α-MSH, activate a significant population of neurons in the PVN to increase autonomic output [33], [35] and inhibit food intake [36]. While the present data indicate that only a fraction of POMC neurons terminate in the PVN, it may be that this relatively sparse innervation is sufficient to affect a large population of cells. For example, in a previous study α-MSH terminals were found on only 30% of thyrotropin-releasing-hormone (TRH) neurons in the medial parvocellular PVN, yet all TRH neurons in the region showed increased TRH mRNA with α-MSH treatment [37]. Thus, α-MSH appears capable of mediating a potent physiological response in more cells than are directly innervated by POMC terminals.

Both β-end and α-MSH affect the activity of neurons in the SON, particularly oxytocin-releasing neurons [38], [39]. The present results are in line with previous work demonstrating retrograde labeling from the SON to the arcuate nucleus and in a subset (∼20%) of POMC neurons [34]. Similarly, POMC neurons innervate selective neurons in the LH including those that express orexin or melanin-concentrating hormone [33], [40], [41]. While it is difficult to compare the results from previous studies with those here quantatively due to the variety of tracers and animal species used, it is clear that only a subset of POMC neurons innervates each of the hypothalamic target sites.

### Extrahypothalamic targets of POMC neurons

POMC neuron projections to the VTA, PAG, BST, and amygdala have been demonstrated in immunohistochemical studies and by measuring POMC peptides in these target regions [31]. The retrograde labeling approach used here supports the presence of POMC terminals in these extrahypothalamic regions. Interestingly, despite little evidence of POMC-peptide containing fibers in the VTA, injections of CTB into the VTA labeled approximately the same percentage of POMC cells as when the tracer was injected into hypothalamic nuclei (Figure 4). The presence of functional POMC terminals in the VTA was previously indicated by the ability of melanocortin receptor antagonists injected into the VTA to decrease behavioral responses [42] and dopamine release [43]. Thus, the ∼8% of POMC cells that project to the VTA under basal conditions must release a sufficient amount of transmitter to evoke physiologic responses.

Although tracer injections into the PAG or BST labeled approximately the same number of total cells in the arcuate nucleus as injections into the VTA and hypothalamic nuclei (Figure 4), relatively fewer of the labeled cells were POMC neurons (Figure 5) suggesting that these regions receive innervation from a very small number of POMC neurons. Despite the small number of POMC neurons that appear to project to areas like the PAG and BST, significant β-end and α-MSH release has been noted in these regions [11], [13], [44], and POMC peptides acting at receptors in the PAG and BST are suggested to play important roles in analgesia and hypothalamus-pituitary-adrenal axis activation, respectively [45], [46], [47]. Therefore, it is feasible that the innervation from relatively few POMC neurons is sufficient to mediate physiologic responses of POMC peptides in these areas.

POMC projections to the amygdala were previously indicated by the presence of β-endorphin immunoreactive fibers [11] and release of POMC peptides [13], [48], [49] into the amygdala. The present results indicate only ∼2% or less of POMC neurons in the arcuate nucleus project to the amygdala (Figure 4) under basal conditions. The low number of POMC neurons projecting to the amygdala is somewhat surprising given the important role of POMC peptides in this area to mediate aspects of appetite and affective state [50], [51], [52]. However, it is possible that POMC neurons innervate a part of the amygdala that was not targeted in the current study.

Melanocortin receptors (MC4) are heavily expressed in the dorsal vagal complex (DVC, 10N and NTS) [53], [54]. The release of POMC peptides into the hindbrain affects food intake and body weight [55] and induces hypotension and bradycardia [56]. Stimulation studies [56] and recent retrograde labeling studies [21] clearly indicate that arcuate nucleus POMC neurons mediate actions in the DVC consistent with the present data indicating POMC projections to the DVC. The low percent of POMC neurons projecting to the DVC is in agreement with a previous report where the authors found that the majority of POMC neurons that project to the DVC reside in the rostral portion of the arcuate nucleus [57]. Therefore, POMC neurons projecting to the DVC may represent an anatomically and functionally distinct subset of POMC neurons with sparse but important projections to the DVC.

### Heterogeneity of POMC neurons

Several reports suggest that POMC neurons in different portions of the arcuate nucleus have distinct characteristics. For example, (1) estradiol increases *Pomc* mRNA to a greater extent in the rostral arcuate nucleus than more caudally [58]. (2) Adrenalectomy decreases *Pomc* in more caudal cells [59]. And (3) the rostral, but not caudal-most, POMC cells are activated by leptin administration [60]. In addition to differences in the regulation of POMC neurons, it has been proposed that the release of co-transmitters from POMC neurons may vary across the rostral-caudal extent of the arcuate nucleus [8]. Finally, there is not always a 1∶1 ratio of each of the POMC-derived peptides released into terminal regions, as processing of the POMC prohormone can vary in individual cells resulting in differential release of peptides in specific target sites [61], [62], [63]. Given this heterogeneity, it was expected that different subsets of POMC neurons would project to select target sites. Similar to previous studies, the results here show that POMC neurons projecting to the DVC and PAG are more likely to reside in the rostral portion of the arcuate nucleus ([57], [64] and Figure 6). However, most target sites appear to be innervated by POMC cells dispersed throughout the arcuate nucleus. Nonetheless, the results to date do not rule out the possibility that POMC neurons projecting to individual target sites may be specialized in some way that is not reflected by location alone such as expression of select receptors or co-expression of specific transmitters.

### Technical considerations

All tracers used for anatomic mapping of neuronal fibers have some advantages, disadvantages and limitations [65]. The majority of the present studies were performed using CTB as the tracer because its utility as a retrograde tracer has been demonstrated in numerous papers since its early use in hypothalamic tracing studies [66]. Although anterograde labeling can also be performed with CTB [67], without amplified immunodetection the anterograde signal is very weak and does not limit the ability to detect the strong label in the soma resulting from retrograde transport [67], [68]. Since there is some suggestion that CTB may be taken up by fibers of passage, some experiments were replicated using fluorescent microspheres which are not taken up by fibers of passage [65]. No difference was detected in the number of POMC cells labeled using these two tracers suggesting CTB uptake by axon terminals selectively, although both tracers may be taken up by damaged fibers [65]. Given the low level of retrograde labeling observed and the consistency of the current results with previous studies, it seems unlikely that damaged fibers are contributing significantly to CTB labeling seen here. Additionally, if the injection was causing significant damage and CTB was taken up by damaged fibers, then doubling the injection volume should increase the amount of labeling observed which did not occur, supporting selective uptake by axon terminals. Whereas false-positive labeling seems unlikely, it is possible that absolute the number of cells labeled with tracer is an underestimate since injections were made unilaterally.

Finally, the low number of labeled cells together with marked variability between subjects limits the ability to detect small differences between target sites or among different regions of the arcuate nucleus. As indicated by the 95% confidence intervals only relatively large differences in the percent of POMC cells with CTB could be detected. Increasing the number of animals tested was not further pursued since the total number of POMC cells with retrograde label was consistently less than 10%. Thus even with better statistical power, the physiologic significance of differences in location among 10% or less of the POMC neuron population would be difficult to interpret.

### Conclusions

POMC neurons from the arcuate nucleus send projections widely throughout the brain to affect an array of functions. The current results suggest that only a small number of POMC neurons terminate in any individual target site, thus relatively sparse innervation may be sufficient to induce the physiologic responses attributed to POMC peptide actions in specific brain regions although contributions of brainstem POMC neurons cannot be ruled out. Further studies will need to examine whether the innervation of targets is plastic and if more POMC neurons can be recruited to project to targets in a state-dependent manner. In the absence of such plasticity, the data here suggest that disturbed function of very few POMC neurons could have significant consequences depending on the target site those neurons innervate. Future studies that combine tract-tracing and functional analyses together will provide much needed insight into the plasticity and specificity of innervation of target sites by POMC neurons.
